# MYC promotes fibroblast osteogenesis by regulating ALP and BMP2 to participate in ectopic ossification of ankylosing spondylitis

**DOI:** 10.1186/s13075-023-03011-z

**Published:** 2023-02-21

**Authors:** Qianmei Jin, Yaoyang Liu, Zhiguo Zhang, Xingzhu Wen, Ziqiang Chen, Haijun Tian, Zijian Kang, Xin Wu, Huji Xu

**Affiliations:** 1 Department of Rheumatology and Immunology, Shanghai Changzheng Hospital, Naval Medical University, Shanghai, 200003 China; 2 Department of General Surgery, 72nd Group Army Hospital, Huzhou University, Huzhou, 313000 Zhejiang China; 3 Department of Orthopaedics, Changhai Hospital, Naval Medical University, Shanghai, 200433 China; 4 Shanghai Key Laboratory of Orthopaedic Implants, Department of Orthopaedic Surgery, Shanghai Ninth People’s Hospital, Shanghai Jiao Tong University School of Medicine, Shanghai, 200025 China; 5 School of Medicine, Tsinghua University, Beijing, 100084 China; 6 Peking-Tsinghua Center for Life Sciences, Tsinghua University, Beijing, 100084 China

**Keywords:** Ankylosing spondylitis, Inflammation, Ligaments, Ectopic ossification, MYC

## Abstract

**Background:**

Ectopic ossification is an important cause of disability in patients with ankylosing spondylitis (AS). Whether fibroblasts can transdifferentiate into osteoblasts and contribute to ossification remains unknown. This study aims to investigate the role of stem cell transcription factors (POU5F1, SOX2, KLF4, MYC, etc.) of fibroblasts in ectopic ossification in patients with AS.

**Methods:**

Primary fibroblasts were isolated from the ligaments of patients with AS or osteoarthritis (OA). In an in vitro study, primary fibroblasts were cultured in osteogenic differentiation medium (ODM) to induce ossification. The level of mineralization was assessed by mineralization assay. The mRNA and protein levels of stem cell transcription factors were measured by real-time quantitative PCR (q-PCR) and western blotting. MYC was knocked down by infecting primary fibroblasts with lentivirus. The interactions between stem cell transcription factors and osteogenic genes were analysed by chromatin immunoprecipitation (ChIP). Recombinant human cytokines were added to the osteogenic model in vitro to evaluate their role in ossification.

**Results:**

We found that MYC was elevated significantly in the process of inducing primary fibroblasts to differentiate into osteoblasts. In addition, the level of MYC was remarkably higher in AS ligaments than in OA ligaments. When MYC was knocked down, the expression of the osteogenic genes alkaline phosphatase (ALP) and bone morphogenic protein 2 (BMP2) was decreased, and the level of mineralization was reduced significantly. In addition, the ALP and BMP2 were confirmed to be the direct target genes of MYC. Furthermore, interferon-γ (IFN-γ), which showed high expression in AS ligaments, was found to promote the expression of MYC in fibroblasts in the process of ossification in vitro.

**Conclusions:**

This study demonstrates the role of MYC in ectopic ossification. MYC may act as the critical bridge that links inflammation with ossification in AS, thus providing new insights into the molecular mechanisms of ectopic ossification in AS.

**Supplementary Information:**

The online version contains supplementary material available at 10.1186/s13075-023-03011-z.

## Background

Ankylosing spondylitis (AS) is a highly heritable chronic inflammatory disease that primarily affects the spine and pelvis [1–3]. It causes pain and initially reversible stiffness, ultimately leading to joint ankylosis due to ectopic ossification [4, 5]. Ectopic ossification can result in the loss of joint mobility and ankylosis, leading to severe disability. Despite the clinical impact of ankylosis and its consequences of pain and negative lifestyle changes in patients, the mechanisms of ectopic ossification in AS remain largely unknown [6, 7].

Osteoblasts are the primary cells involved in ectopic ossification [8]. It has been reported that subchondral granulation tissue carries osteoblasts leading to intraarticular ankylosis of the facet joints in AS [9]. The upregulation of calcium-sensing receptors induced by various inflammatory cytokines could promote ossification in AS in osteoblasts [10]. However, these studies did not address the progenitors of osteoblasts, which are essential for the process of ossification.

Earlier studies have indicated that osteoblasts may originate from mesenchymal stem cells (MSCs) [11, 12]. Bone morphogenic protein 2 (BMP2) overexpression in AS-MSCs led to enhanced osteogenic differentiation by activating the Smad1/5/8 and ERK signalling pathways [13]. However, fibroblasts, which are the primary cells in ligament tissue, also showed high BMP2, TGF-β1 and TβRIII expression and auto-osteogenic capacity in the AS spinal supraspinous ligament [14]. It has also been reported that fibroblasts show the potential to differentiate into osteoblasts under specific circumstances in vitro [15, 16]. All of the above research supports the possibility that the fibroblasts may trans-differentiate into osteoblasts to participate in ectopic ossification in AS. Thus, the mechanisms of transdifferentiation merit exploration.

The discovery that somatic cells can be reprogrammed to induced pluripotent stem (iPS) cells by merely four transcription factors (POU5F1, SOX2, KLF4, and MYC) was an important breakthrough in stem cell research [17]. These four transcription factors play an essential role in maintaining stemness, dedifferentiation, and redifferentiation. A study revealed that mouse embryonic fibroblasts could be transdifferentiated into a wide range of somatic lineages in vitro [18]. It was also reported that MYC and KLF4 could convert murine fibroblasts into osteoblasts in vitro, and overexpression of MYC strongly promoted osteoblast differentiation [19, 20]. However, whether these transcription factors play a role in the transdifferentiation of fibroblasts to osteoblasts in AS remains unknown. In the presence of tumour necrosis factor (TNF), astrocytes were able to dedifferentiate by inducing the expression of POU5F1 through the NF-κB pathway, indicating an association between reprogramming and inflammation [21].

Inflammation is an early characteristic of AS [22, 23], but how inflammation proceeds to ossification remains unknown. Since stem cell transcription factors play a critical role in cell reprogramming, whether transcription factors link inflammation with new bone formation in AS is worth addressing.

Thus, we hypothesized that resident fibroblasts in ligaments could be transdifferentiated to osteoblasts via pivotal transcription factors in the inflammatory environment, which may contribute to ectopic ossification in AS. To test this hypothesis, we first screened the transcription factors in both the osteogenic model and ligament samples and then knocked down the specific transcription factor to verify its possible role in transdifferentiation. Then, a chromatin immunoprecipitation (ChIP) assay was performed to test the relationship between the transcription factor and osteogenic genes. Finally, we explored the potential inflammatory cytokines that may be involved in fibroblast transdifferentiation.

## Methods

### Patients and samples

In total, 12 AS and 12 osteoarthritis (OA) patients who underwent a hip replacement procedure were recruited in this study (Additional Table 1). These patients’ ligament samples were collected after the surgical procedure. AS diagnosis was based on New York-modified criteria [24]. The study was conducted in accordance with the principles expressed in the Declaration of Helsinki and was approved by the ethics committee of Shanghai Ninth People’s Hospital, Shanghai Jiao Tong University School of Medicine (SH9H-2019-T177-2). Informed consent was obtained from all patients.

### Ligaments and cells

The ligaments obtained from the hip replacement operation were cut into small fragments of approximately 5 mm^3^ in size. Some fragments were plated in a 100-mm dish with fresh DMEM (Corning, 10–013-CVR) and 10% foetal bovine serum [FBS] (Sigma, F141) to isolate the primary fibroblasts. Fibroblasts between 3 and 8 passages were used in the experiments.

### Primary fibroblast identification

Immunohistochemistry (IHC) and flow cytometry were performed for fibroblast verification. Vimentin (Abcam, ab92547, clone EPR3776), CD29 (BioLegend, 303,007, clone, TS2/16) and CD90 (BioLegend, 328,109, clone 5E10) were used to identify the fibroblasts. In addition, we excluded haematopoietic lineage cells positive for CD45 (BioLegend, 304,006, clone HI30), mesenchyme stem cells and vascular endothelial cells positive for CD106 (BioLegend, 305,805. clone STA) [25, 26].

### Osteogenic model in vitro 

The osteogenic differentiation medium (ODM) consisted of α-MEM (HyClone, SH30265.01) with 10% FBS, 100 nM dexamethasone (Sellack, S1322), 3.5 mM β-glycerophosphate (Stemcell, 05,406) and 50 µg/ml ascorbic acid (Sigma, A4544). The control medium (CON) consisted of α-MEM with 10% FBS.

### Knockdown of the MYC gene in vitro 

shRNA targeting human MYC and a nonspecific scramble shRNA were cloned into the lentiviral vector U6-Cherry-Puromycin. The fibroblasts were exposed to the lentivirus at a multiplicity of infection (MOI) of 20 once they reached 50% confluence. Polybrene (10 µg/ml) was added to enhance the infection efficiency. After 72 h, fibroblasts were selected by culture with complete DMEM and 4 µg/ml puromycin for 48 h.

### Reverse transcription polymerase chain reaction (RT-PCR) and real-time quantitative PCR (q-PCR) analyses

Total RNA was extracted with an RNA fast2000 kit (Fastagen, 220,011). The complementary DNA was synthesized with PrimeScript™ RT Master Mix (Takara, 036A). SYBR Green (Takara, 820A) was used following the manufacturer’s instructions for quantitative analyses. GAPDH was used as the reference gene. The relative expression in the tissues was calculated using 2^−(∆Ct)^. The fold changes in CON and ODM or cytokine stimulation were calculated using 2^−(∆∆Ct)^. The primer sequences are listed in Additional Table 2.

### Western blot

The frozen ligament tissue was crushed by steel balls and lysed with RIPA buffer. The protein concentration was detected by a BCA kit (Beyotime, P0010). Equal amounts of proteins were loaded onto precast 10% SDS-PAGE criterion gels (GeneScript, M00664) and transferred onto polyvinylidene fluoride membranes. The membranes were blocked with 5% nonfat milk at room temperature for 2 h and then incubated with anti-MYC (Millipore, 06–340) or anti-GAPDH (Cell Signaling Technology, 2118 s) overnight at 4 °C. After washing three times, the membranes were incubated with anti-mouse or anti-rabbit antibodies (Cell Signaling Technology, 7076S and 7074S) for 1 h at room temperature. The resulting membranes were detected via the enhanced chemiluminescence method and quantified using ImageG.

### Alkaline phosphatase (ALP) staining

ALP was stained with a BCIP/NBT kit (Beyotime, C3206). First, the cells were washed 3 times with PBS and fixed with ethanol for 20 min. Then, the fibroblasts were washed 3 times again and incubated with the ALP dye solution for 1 to 4 h. Finally, the solution was replaced with distilled water, and photographs were taken.

### Mineralization staining

The level of mineralization was assessed by an OsteoImage™ Mineralization Assay (Lonza, PA-1503). The cells were first fixed with ethanol for 20 min, rinsed 1–2 times with wash buffer, incubated in the dark with staining reagent for 30 min at room temperature, and washed 3 times with wash buffer for microscope viewing.

### Cytokine stimulation

Primary fibroblasts were treated with 100 ng/ml recombinant human interleukin-17 (IL-17), interleukin-22 (IL-22), interleukin-23 (IL-23), TNF-α and interferon-γ (IFN-γ) in the presence of CON or ODM. Then, the cells were collected for q-PCR analysis.

### ChIP

A ChIP assay was performed according to the manufacturer’s protocols (Millipore, 17–10,085). Briefly, the primary fibroblasts were cultured in ODM for 20 days and cross-linked with 1% formaldehyde for 10 min. The cells were lysed with lysis buffer. DNA was fragmented to 200 ~ 1000 bp using a sonicator (Tomy Seiko Co., UR-20P) (Additional Fig. 1). Then, 1% of the chromatin was removed and used as the input control. Normal mouse IgG and an MYC antibody (Millipore, 06–340) were used for immunoprecipitation [27]. Purified DNA was further used for q-PCR. The fold enrichment of the target genes was calculated against mouse IgG.

### IHC

Immunohistochemistry analysis of the paraffin-embedded ligaments was performed to detect MYC-, IL-23- and IFN-γ-positive cells using an anti-MYC antibody (ZSGB-BIO, ZA-0555, clone, EP121), anti-IL-23 antibody (Abcam, ab45420, polyclone) and IFN-γ antibody (Abcam, ab9657, polyclone). For quantification of MYC-, IL-23- or IFN-γ-positive cells, the cells with nuclear or cytoplasmic signals in high-power fields (hpf) were counted.

### Data analysis

An unpaired nonparametric test was used for the tissue data. A paired parametric *t* test was used for paired comparisons. Two-way analysis of variance (ANOVA) followed by Tukey’s post hoc test and multiple *t* test was used for multiple comparisons. The Wilcoxon matched-pairs signed rank test was used for short-term cytokine stimulation. Data used for analysis were from at least three independent tests. A *P* value < 0.05 was considered significant. Parametric data are shown as the mean ± standard error of the mean (SEM).

## Results

### MYC was upregulated in an osteogenic cell model and ligaments from patients with AS

We isolated and identified primary fibroblasts from the hip ligaments of patients with OA and AS, with the detailed data shown in Additional Fig. 2 . To simulate the dynamic osteogenic process and assess the changes in stem cell transcription factors, a classic in vitro osteogenic model was used to induce fibroblasts to ossification [28].

**Fig. 1:**
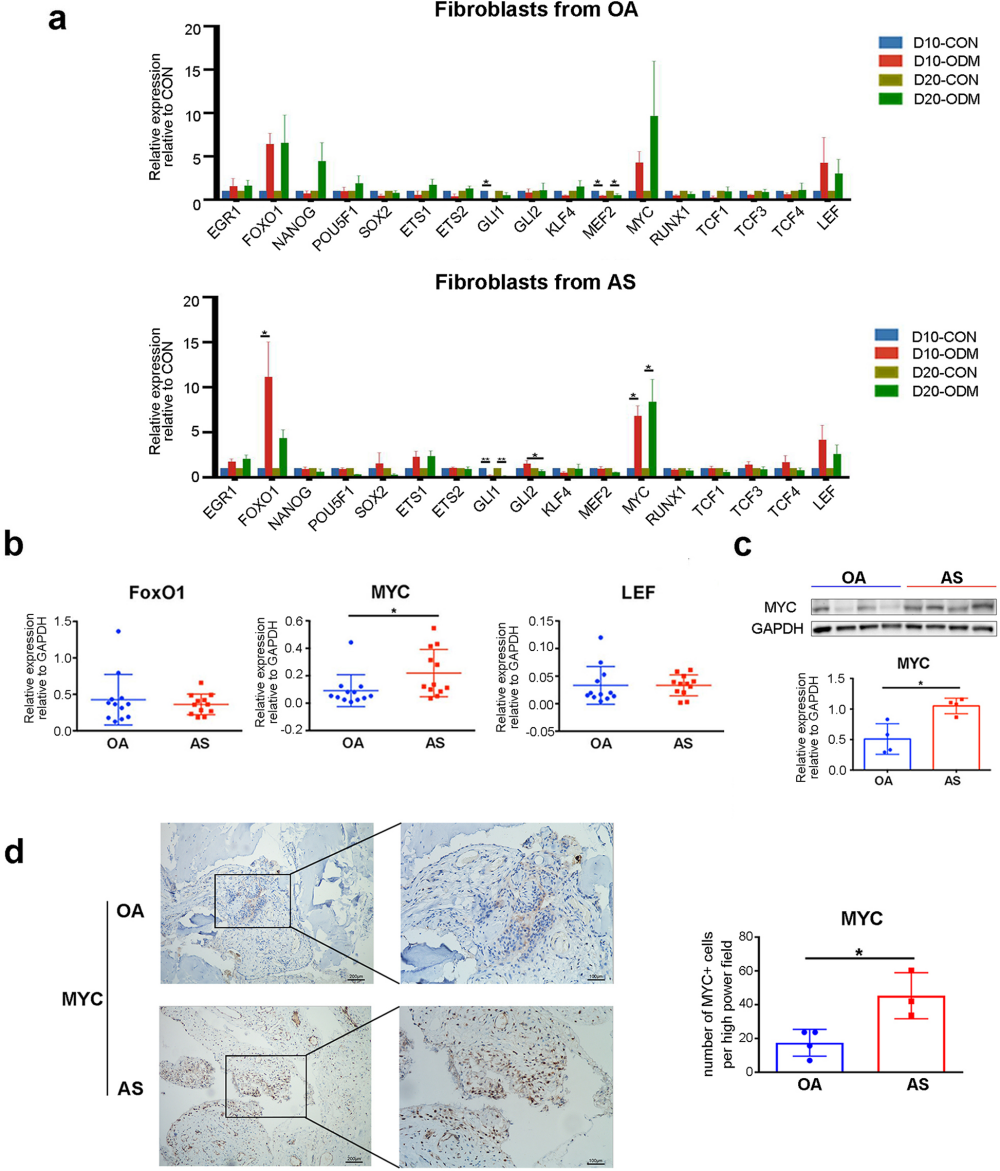
MYC was upregulated in an osteogenic cell model and hip ligaments in patients with AS. **a** Fibroblasts from OA (*n* = 3) and AS (*n* = 4) patients were cultured in osteogenic differentiation medium (ODM) or control medium (CON). Stem cell transcription factors were measured by q-PCR on D10 and D20. **b** Upregulated transcription factors were assessed by q-PCR in ligaments from OA (*n* = 12) and AS (*n* = 12) patients. **c** Transcription factor MYC was measured by WB in ligaments from OA (*n* = 4) and AS (*n* = 4) patients. GAPDH was used as the housekeeping gene. The grouping of gels/blots was cropped from different parts of the same gel. No high-contrast (overexposure) of blots was performed. **d** Representative images shown MYC staining in the ligaments of patients with OA (*n* = 4) and AS (*n* = 3). Boxed areas in the left panels are shown at higher magnification in the right panels. Scale bar = 200 µm (left panels). Scale bar = 100 µm (right panels). Values are reported as the mean ± SEM. *p < 0.05

Fibroblasts isolated from the ligaments were cultured in ODM or CON for 10 days or 20 days, and the mRNA levels of classic stem cell transcription factors were measured by q-PCR. As shown in Fig. 1a, FOXO1 was significantly upregulated in AS fibroblasts on D10. MYC was significantly upregulated in AS fibroblasts on D10 and D20. Although the differences were not significant, FOXO1 and MYC were also upregulated in the ODM group in OA fibroblasts, and LEF tended to increase in the ODM groups from both OA and AS fibroblasts. Presumed to be critical in the ‘turn-on’ of the osteogenic gene transcription, the 3 upregulated stem cell transcription factors were further assessed in ligament samples from patients by q-PCR. The results showed that only MYC expression was higher in AS ligaments than in OA ligaments (Fig. 1b). Thus, we further tested the protein level of MYC in ligaments via western blotting and IHC and confirmed the upregulation of MYC in AS samples (Fig. 1c, d). Collectively, MYC was upregulated in both the osteogenic model and AS ligament samples, which indicates its possible role in promoting the transdifferentiation of resident fibroblasts to osteoblasts.

### MYC promoted fibroblast transdifferentiation by upregulating ALP and BMP2

**Fig. 2:**
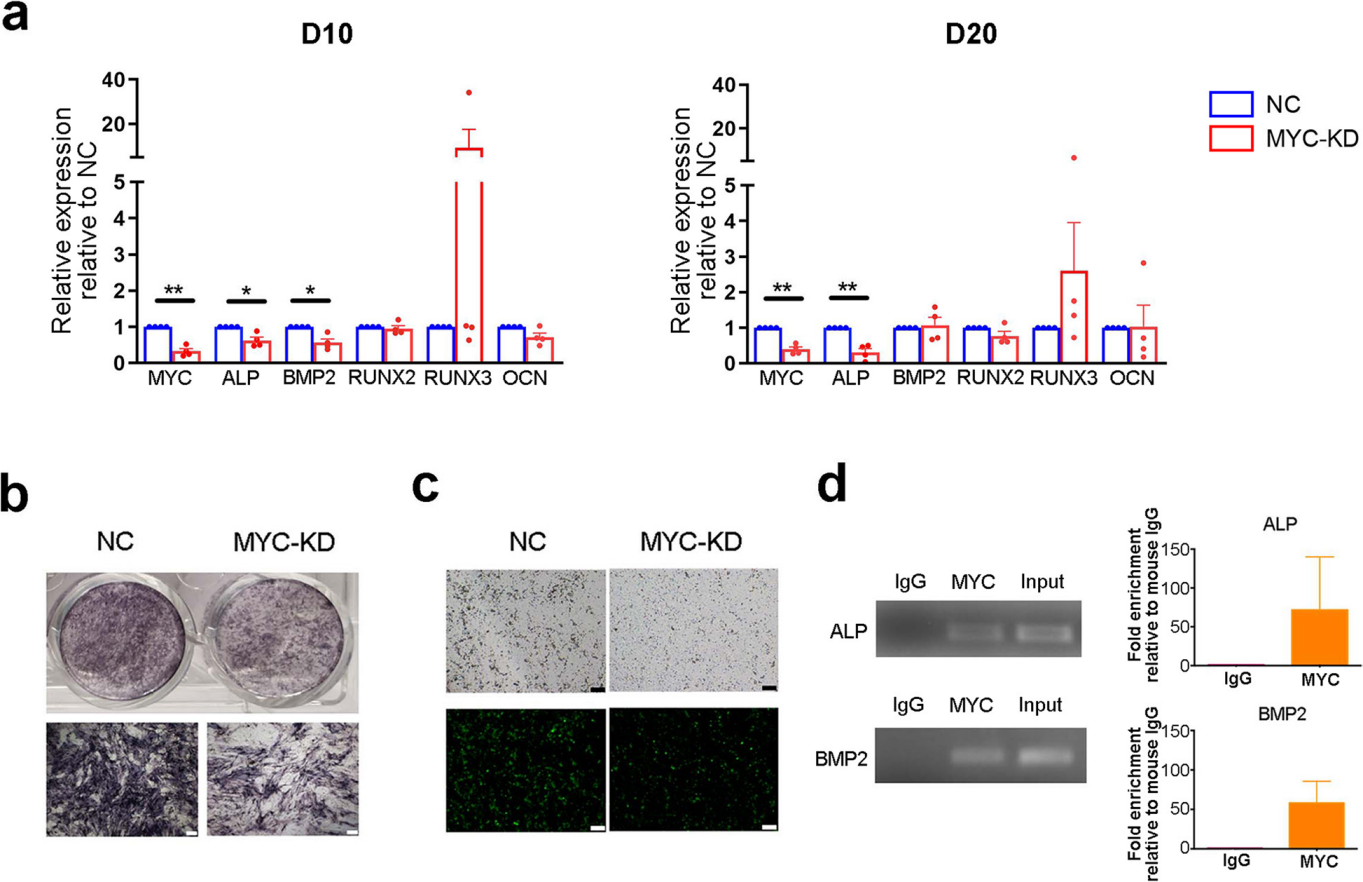
MYC promoted fibroblast transdifferentiation by upregulating ALP and BMP2. **a** Fibroblasts were infected with negative control lentivirus (NC) and MYC knockdown lentivirus (MYC-KD) and cultured in ODM for 10 or 20 days. The expression levels of the transcription factor MYC and osteogenic genes were measured by q-PCR (*n* = 4). **b** ALP was stained in fibroblasts cultured in ODM from NC and MYC-KD groups on D20, shown in the culture dish (upper panel) and under the microscope (lower panel). Scale bar = 200 µm. **c** Levels of mineralization in fibroblasts were measured in NC and MYC-KD groups on D20 by OsteoImageTM Mineralization Assay, shown in light field (upper panel) and fluorescent light (lower panel) microscopically. Scale bar = 100 µm. **d** The interactions between the transcription factor MYC and osteogenic genes were confirmed by ChIP, and representative gels of the amplified promoters of ALP and BMP2 are shown in the left panel. The grouping of gels/blots was cropped from different gels. No high contrast (overexposure) of blots was performed. Fold enrichment of ALP and BMP2 was calculated over negative control mouse IgG (right panel). Values are reported as the mean ± SEM. *p < 0.05. **p < 0.01

To explore the possible mechanism of transdifferentiation, MYC was knocked down by infecting primary fibroblasts (from AS) with MYC knockdown lentivirus (MYC-KD). The control group was infected with a negative control lentivirus (NC). Both groups were cultured in ODM for 10 days or 20 days. The osteogenic genes ALP, BMP2, Runt-related transcription factor 2 (RUNX2), Runt-related transcription factor 3 (RUNX3) and osteocalcin (OCN) were detected by qPCR to evaluate the effect of MYC on ossification. As shown in Fig. 2a, ALP and BMP2 showed significantly lower expression on D10 when MYC was knocked down. Notably, ALP continued to show significantly lower expression on D20 in the MYC-KD group. Then ALP in fibroblasts was stained and compared between the NC and MYC-KD groups. As shown in Fig. 2b, the level of ALP was lower in the MYC-KD group. Consistently, the degree of mineralization was dominantly lower in the MYC-KD group than in the NC group (Fig. 2c). Taken together, the above results indicated that MYC may play an important role in fibroblast transdifferentiation, and ALP and BMP2 are the target genes of MYC.

To verify the above hypothesis, the relationship between MYC and osteogenic genes was further analysed in fibroblasts by ChIP assay in ODM for 20 days. As shown in Fig. 2d, a remarkably larger amount of chromatin containing the ALP and BMP2 promoters was immunoprecipitated by the anti-MYC antibody than by the negative control IgG. ALP’s fold enrichment was approximately 70 times and that of BMP2 was approximately 60 times, which strongly supported that MYC could bind the promoters of ALP and BMP2. These results suggested that MYC may contribute to the transdifferentiation of fibroblasts to osteoblasts by regulating the osteogenic genes ALP and BMP2, which may be involved in the mechanism of ectopic ossification in AS.

### IFN-γ promoted fibroblast transdifferentiation by regulating MYC in AS

**Fig. 3:**
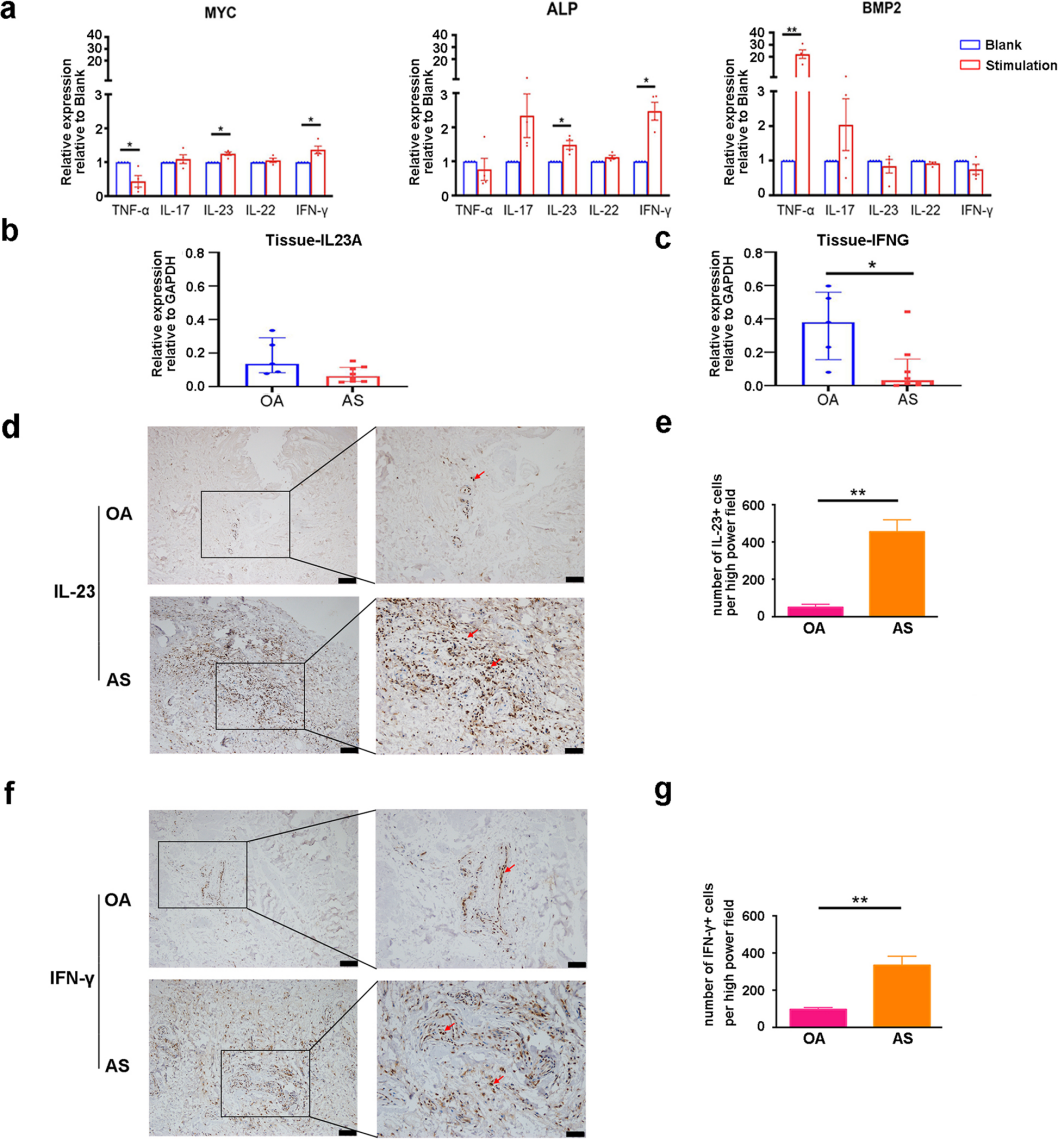
IL-23 and IFN-γ showed higher frequencies in AS ligaments than in OA ligaments. **a** Primary fibroblasts were treated with 100 ng/ml recombinant human cytokines for 24 h. Stem cell transcription factors and osteogenic marker genes were measured with q-PCR (*n* = 4). **b** The relative expression of IL23A at the mRNA level in OA (*n* = 5) and AS (*n* = 8) ligament samples. **c** The relative expression of IFNG at the mRNA level in OA (*n* = 5) and AS (*n* = 8) ligament samples. **d** Representative images shown IHC staining for IL-23 in the ligaments of patients with AS and OA. Boxed areas in the left panels are shown at higher magnification in the right panels. Positive cells (arrows). Scale bar = 100 µm (left panels). Scale bar = 50 µm (right panels). **e** Quantification of IL-23-positive cells is displayed (*n* = 3). **f** Representative images shown IHC staining for IFN-γ in the ligaments of patient with AS and OA. Boxed areas in the left panels are shown at higher magnification in the right panels. Positive cells (arrows). Scale bar = 100 µm (left panels). Scale bar = 50 µm (right panels). **g** Quantification of IFN-γ-positive cells is displayed (*n* = 3). Values are reported as the median ± SEM. *p < 0.05. **p < 0.01

Given that inflammatory cytokines play an important role in the pathogenesis of AS, it is of great interest to explore their possible role in the process of ossification. To evaluate the role of cytokines in regulating MYC, ALP and BMP2, the primary fibroblasts were treated with recombinant human TNF-α, IL-17, IL-23, IL-22 and IFN-γ for 24 h in vitro, respectively. As shown in Fig. 3a, IL-23 and IFN-γ significantly upregulated the mRNA expression level of MYC in the fibroblasts. Consistently, the level of ALP was also upregulated in the presence of IL-23 and IFN-γ. These findings suggested that IL-23 and IFN-γ may promote fibroblast transdifferentiation into osteoblast via MYC. In addition, we found that TNF-α significantly decreased MYC levels and upregulated BMP2 levels in the fibroblasts. This result indicated that there may be other mechanisms by which TNF-α to regulates ossification in fibroblasts.

We further verified the expression of IL-23 and IFN-γ at the mRNA and protein levels in ligament samples from patients with OA and AS. As shown in Fig. 3b, there was no significant difference of the relative expression of IL23A between AS and OA samples. The relative expression of IFNG was significantly lower in AS samples. However, a significantly higher frequency of IL-23-positive cells in AS ligaments (mean ± SEM, 454.3 ± 67.8/hpf) than in OA ligaments (mean ± SEM, 50.4 ± 17.1/hpf) and a significantly higher frequency of IFN-γ-positive cells in AS ligaments (mean ± SEM, 333.7 ± 49.1/hpf) than in OA ligaments (mean ± SEM, 96.0 ± 10.6/hpf) were observed (Fig. 3d–g). The inconsistent trends between mRNA and protein levels may be due to negative feedback inhibition after IL-23 and IFN-γ overexpression. Collectively, the increased protein levels of IL-23 and IFN-γ indicated a more severe inflammatory response in AS ligaments and their potential role in the ectopic ossification.

**Fig. 4:**
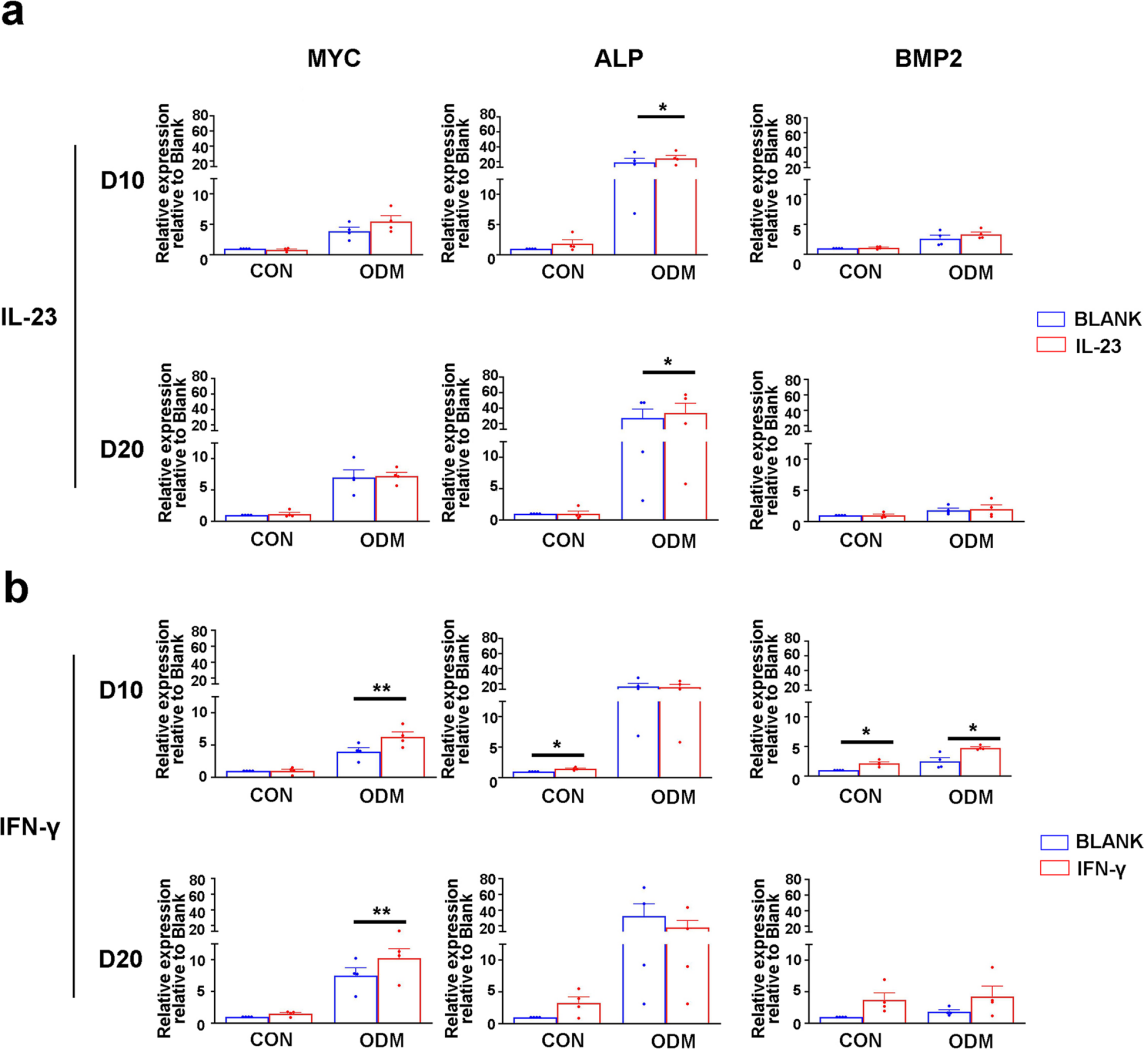
IFN-γ promoted fibroblast transdifferentiated to osteoblasts via MYC in AS. **a**, **b** Primary fibroblasts were treated with 100 ng/ml recombinant human IL-23 and IFN-γ for 10 or 20 days in the presence of CON or ODM. Stem cell transcription factors and osteogenic marker genes were measured with q-PCR (*n* = 4). *p < 0.05. **p < 0.01

Next, IL-23 and IFN-γ were added to the ossification model to verify their role over a longer culture time, which may imitate the chronic inflammatory environment. In the presence of IL-23, only ALP increased in the ODM group on both D10 and D20 (Fig. 4a). In the presence of IFN-γ, the levels of MYC (in ODM), ALP (in CON) and BMP2 (in CON and ODM) were significantly upregulated on D10, and only the level of MYC (in ODM) was significantly upregulated on D20 (Fig. 4b). In brief, IL-23 could only increase the level of ALP, while IFN-γ was able to upregulate MYC, ALP and BMP2. In summary, these results indicated that IFN-γ may play a more important role in promoting fibroblast transdifferentiation into osteoblasts via MYC and thus promote ectopic ossification in patients with AS.

## Discussion

**Fig. 5:**
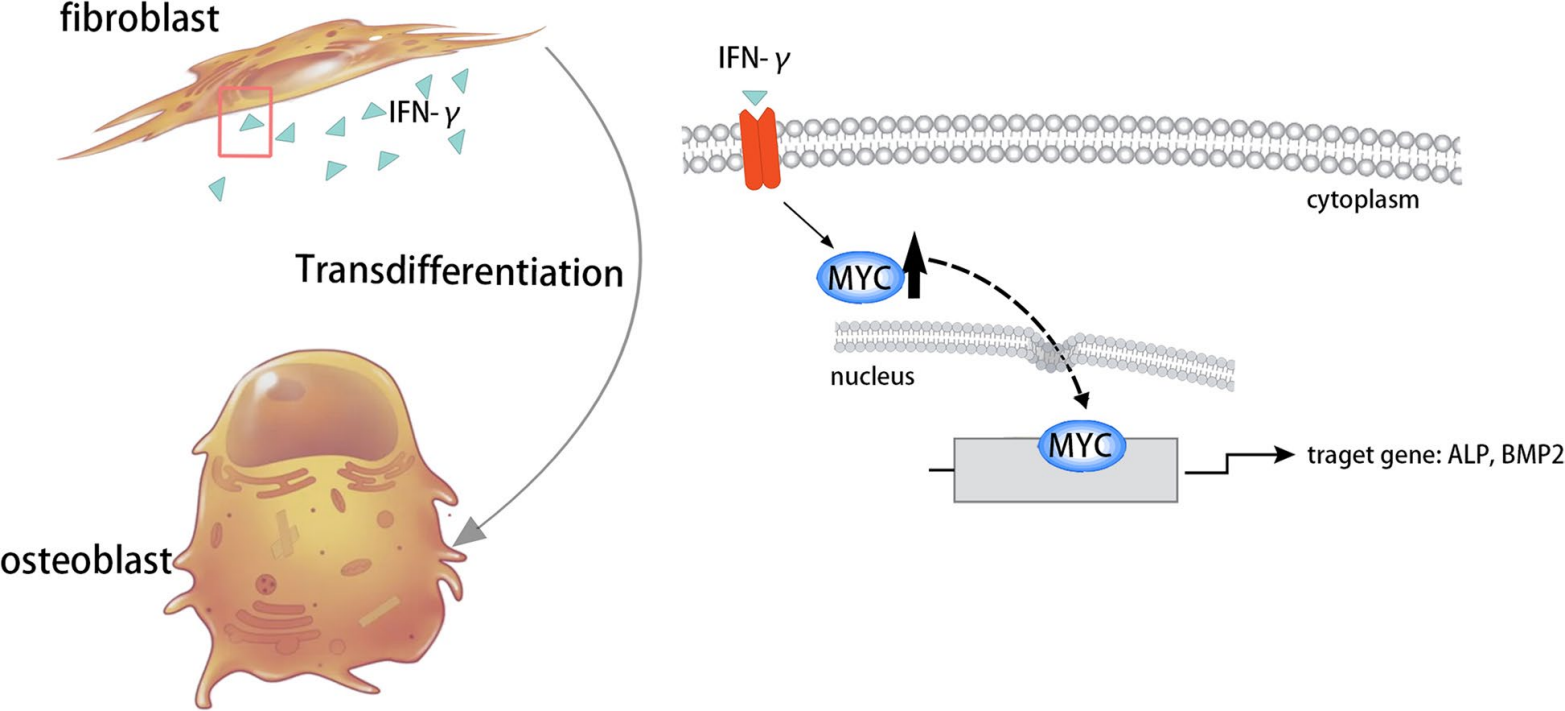
Possible role of MYC in fibroblasts during ectopic ossification in AS. IFN-γ may upregulate the expression of MYC in fibroblasts, and MYC further binds the promoters of ALP and BMP2 to induce their expression, resulting in fibroblast transdifferentiation to osteoblasts and ligament ossification

We hypothesized that fibroblasts in ligaments could be transdifferentiated to osteoblasts via pivotal transcription factors after long-term inflammatory stimulation, which would then participate in ectopic ossification in AS patients. In the current study, we found that IFN-γ can upregulate the expression of MYC in fibroblasts, and MYC further binds the promoters of ALP and BMP2 to induce their expression, which results in fibroblasts transdifferentiated to osteoblasts and ligament ossification (Fig. 5).

Our data supported previous findings that MYC also serves as a target gene of the Wnt signalling pathway, which is the classic pathway involved in osteogenesis and that the downregulation of Dickkopf-1 (DKK1), a Wnt inhibitor, in AS fibroblasts by shRNA silencing could result in the upregulation of MYC [29]. On the one hand, MYC has already been proven to be a key regulator of osteoblast differentiation. Previous studies reported that the doxycycline-inducible expression of MYC and KLF4 could convert murine fibroblasts into osteochondrogenic cells and that the overexpression of MYC strongly promoted osteoblast differentiation [19, 20]. On the other hand, MYC also plays an important role in osteoclast differentiation, since MYC has been reported to be strongly upregulated in RANKL-induced osteoclast-like cells [30]. These multiple roles of MYC suggest that more studies are needed to elucidate its function.

The mammalian RUNX protein family comprises three transcription factors—RUNX1, RUNX2, and RUNX3. RUNX1 is involved in haematopoiesis, RUNX2 has multiple roles in osteogenesis and RUNX3 is associated with neural and gut development [31]. Runx2 is the osteogenic master regulator that integrates signals from various developmental cues [32]. However, an increasing number of studies have reported that RUNX3 is also involved in osteogenesis. The Runx3-deficient mice develop severe congenital osteopenia, and the rs6600247 AS risk allele, located in an enhancer-like region upstream of the RUNX3 promoter, modulates c-MYC binding [33, 34]. However, there was no significant difference in the level of RUNX3 between the MYC-KD and NC groups in this study, indicating that MYC may regulate the transdifferentiation of fibroblasts by some other mechanisms.

Since AS is an immune-mediated inflammatory disorder, it is a significant milestone in AS treatment that tumour necrosis factor inhibitors (TNFis) have proven to be effective in alleviating symptoms and reducing disease activity [22, 23, 35]. Recent studies have also found that cytokines, including TNF-α, migration inhibitory factor (MIF), IL-22 and IL-17, can promote bone formation [28, 36–39]. However, some other reports have shown the independence of inflammation and ossification because the majority of vertebral syndesmophytes occur at sites with no previous inflammation [40]. These controversial findings indicate that more efforts are needed to elucidate the mechanism between inflammation and ossification. Several studies have reported that fibroblasts can effectively respond to IL-23 and IFN-γ stimulation. For example, IL-23 may induce joint inflammation and bone destruction by stimulating RANKL expression in fibroblast-like synoviocytes in RA [41]. IFN-γ had a greater effect on inhibiting cell proliferation by Down syndrome-derived fibroblast cells [42]. These studies supported that fibroblasts may express IL-23 receptors (or IFN-γ receptors) in response to stimulation. Thus, we explored the relationship between transcription factors and inflammation and found that IFN-γ could activate the reprogram of fibroblasts to osteoblasts via upregulating MYC. Consistent with our findings, another recent study showed that IFN-γ could regulate the transformation of microglia into dendritic-like cells via the ERK/MYC signalling pathway [43]. Furthermore, IFN-γ was proved to be able to regulate *myc* expression in mouse fibroblasts [44], while MYC could respond to IFN-γ signalling [45].

Although it is not as well known as IL-17, there is a reasonable body of literature to support the pathogenic role of IFN-γ in AS. The expression of HLA–B27 leads to a significant alteration in IFN-γ signalling in antigen-presenting cells in both B27-transgenic rats and spondyloarthritis patients [46]. Notably, the serum level of IFN-γ is significantly higher in AS patients than in normal people [47]. *IFN*-γ rs2430561 and rs2069727 polymorphisms may contribute to the risk of AS by influencing IFN-γ expression, especially in the Chinese population [48, 49]. Interestingly, a recent study identified anatomically distinct fibroblasts with permissive or repressive IFN-γ responses as the key determinant of body-level patterns of lesions in vitiligo [50]. This finding may provide clues that fibroblasts from different sites may have different responses to IFN-γ, which may help to explain why ectopic ossification in AS occurs mainly in some specific ligaments.

It has been shown in other studies that the IL-23/IL-17 pathway plays a significant role in the pathogenesis of AS [51–53]. According to our data, although more IL-23 was found in AS ligaments than in OA ligaments in situ, there was little impact of IL-23 and IL-17 on MYC, possibly because IL-17 enhanced T-cell priming and stimulated other cells to activate the inflammatory cascade at an early stage [54, 55]. It has also been verified that IL-17 promotes osteoclastogenesis by upregulating RANKL expression, while IFN-γ exerts inhibitory effects on osteoclastogenesis [56, 57]. Taken together, these findings further support our results that IFN-γ, rather than the IL-23/IL-17 pathway, had an effect on MYC and osteogenic genes.

Taken together, this study revealed that stem cell transcription factors established a bridge between inflammation and ectopic ossification, and the direct conversion of fibroblasts to osteoblasts enriched the sources of osteoblasts. While osteoblasts originating from MSCs are considered crucial for ossification in general, fibroblasts, as the resident cells in ligaments and entheses, represent a potential cell bank participating in ectopic ossification.

## Conclusions

Our work highlights the significant role of MYC in fibroblast transdifferentiation and the function of IFN-γ as a potentially vital cytokine activating MYC to promote osteogenesis in patients with AS. Hopefully, these results could offer new insights into the mechanism of ectopic ossification and provide a novel treatment strategy for patients with AS.

## Supplementary Information

**Additional file 1: Supplementary file 1.**

**Additional file 2:Supplementary file 2.**

## Data Availability

The datasets used and/or analysed during the current study are available from the corresponding author on reasonable request.

## Abbreviations

ALP: Alkaline phosphatase
AS: Ankylosing spondylitis
BMP2: Bone morphogenic protein 2
ChIP: Chromatin immunoprecipitation
CON: Control medium
DKK1: Dickkopf-1
IFN-γ: Interferon-γ
IHC: Immunohistochemistry
iPS: Induced pluripotent stem
MIF: Migration inhibitory factor
MOI: Multiplicity of infection
MSCs: Mesenchymal stem cells
MYC-KD: MYC knockdown lentivirus
NC: Negative control lentivirus
OA: Osteoarthritis
OCN: Osteocalcin
ODM: Osteogenic differentiation medium
RUNX2: Runt-related transcription factor 2
TNF: Tumour necrosis factor
TNFis: Tumour necrosis factor inhibitors
