# Supplementary material for: MYC promotes fibroblast osteogenesis by regulating ALP and BMP2 to participate in ectopic ossification of ankylosing spondylitis
Source: Arthritis Res Ther. 2023 Feb 21;25:28. doi: 10.1186/s13075-023-03011-z (PMC9942334; doi:10.1186/s13075-023-03011-z)
Supplement: Supplementary file 1 — Additional file 1: Supplementary file 1. [file 13075_2023_3011_MOESM1_ESM.pdf]

Additional File

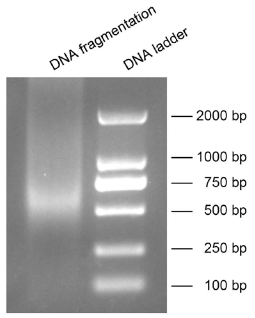

Additional Figure 1. The gel of DNA fragmentation for ChIP assay.

DNA for ChIP assay was fragmented to 200~1000 bp using a sonicator. Left lane is DNA fragmentation. Right lane is DNA ladder.

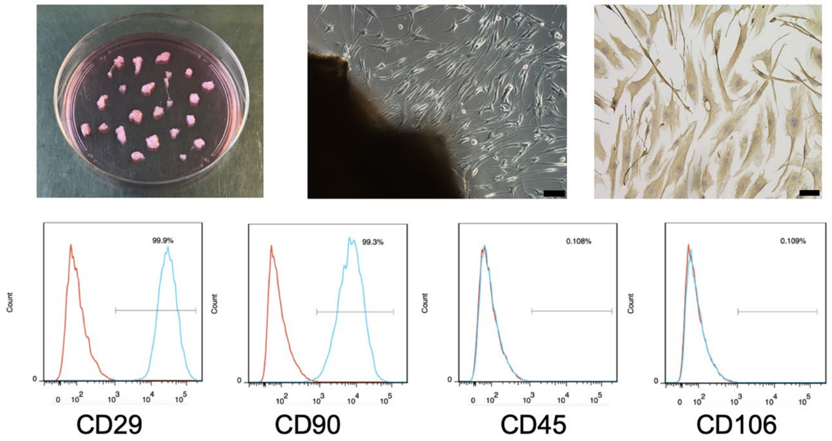

Additional Figure 2. Isolation and indentification of primary fibroblasts.

Ligament tissues were planted in a 100 mm dish (upper left) and primary fibroblasts climbed out around 10 days (upper middle). IHC staining for vimentin (upper right) and a set of surface markers by flow cytometry (lower panels) were used to identify fibroblasts. Scale bar=100 um (upper middle). Scale bar= 50 um (upper right).

Additional Table 1

Patients' Clinical Information

	**OA (n=12)**	**AS (n=12)**
**Age (years)**	50.22±4.19	41.38±4.65
**Gender (Male/Female)**	4/8	12/0
**HLA-B27 (+/-)**	0/12	12/0
**ESR (mm/H)**	10.0 (3.0, 10.5)	18.0 (10.5, 40.5)
**CRP (mg/L)**	3.27 (2.14, 5.60)	14.10 (9.03, 33.00)

Additional Table 2

Primer sequences

Gene	Sequence (5' to 3')
GAPDH-F	ACAACTTTGGTATCGTGGAAGG
GAPDH-R	GCCATCACGCCACAGTTTC
POU5F1-F	GCTGGAGAAGGAGAAGCTGG
POU5F1-R	AAAGCGGCAGATGGTCGTTT
KLF4-F	AGAAGGATCTCGGCCAATTT
KLF4-R	AAGTCGCTTCATGTGGGAGA
SOX2-F	CATCACCCACAGCAAATGAC
SOX2-R	GCAAACTTCCTGCAAAGCTC

Gene	Sequence (5' to 3')
MYC-F	GCTGCTTAGACGCTGGATTT
MYC-R	AGCAGCTCGAATTTCTTCCA
ALP-F	AGCCCTTCACTGCCATCCTGTAT
ALP-R	CGCCTGGTAGTTGTTGTGAGCAT
BMP2-F	ACTACCAGAAACGAGTGGGAA
BMP2-R	GCATCTGTTCTCGGAAAACCT
RUNX2-F	CCAGGCAGTTCCCAAGCATTTCA
RUNX2-R	GGTAGTGAGTGGTGGCGGACATA
OCN-F	CCCTCACACTCCTCGCCCTATT
OCN-R	TCTTCACTACCTCGCTGCCCTC
FOXO1-F	TCGTCATAATCTGTCCCTACACA
FOXO1-R	CGGCTTCGGCTCTTAGCAAA
NANOG-F	GAGATGCCTCACACGGAGACTGT
NANOG-R	TGGGTTGTTTGCCTTTGGGACTG
ETS1-F	TTGAAAGCATAGAGAGCTACGA
ETS1-R	CTCTGAGTCGAAGCTGTCATAG
ETS2-F	GCTCCGTCAGCGTCACCTA
ETS2-R	AACCCGTTGCACATCCAG
GLI1-F	AACCCTTGGAAGGTGATATGTC

Gene	Sequence (5' to 3')
GLI1-R	TTCATACACAGATTCAGGCTCA
GLI2-F	CAAGAAGCCAAAAGTGGGATC
GLI2-R	CAGAATGAGGCTCGTAATGGTA
MEF2-F	ACTACAGACCTCACAGTGCCA
MEF2-R	GCCTAAGCTATTTGCACCAGT
RUNX1-F	TGAGCTGAGAAATGCTACCGC
RUNX1-R	ACTTCGACCGACAAACCTGAG
TCF1-F	TTGATGCTAGGTTCTGGTGTACC
TCF1-R	CCTTGGACTCTGCTTGTGTC
TCF3-F	CTCTCCAACGGACCCCTGT
TCF3-R	TGACCTCGTGTCCTTGACTGT
TCF4-F	AGAAACGAATCAAAACAGCTCCT
TCF4-R	CGGGATTTGTCTCGGAAACTT
LEF-F	TGCCAAATATGAATAACGACCCA
LEF-R	GAGAAAAGTGCTCGTCACTGT
IFNG-F	TCGGTAACTGACTTGAATGTCCA
IFNG-R	TCGCTTCCCTGTTTTAGCTGC
IL23A-F	CTCTGCTCCCTGATAGCCCT
IL23A-R	TGCGAAGGATTTTGAAGCGG

Gene	Sequence (5' to 3')
RUNX3-F	GCGAGGGAAGAGTTTCACCC
RUNX3-R	TTGATGGCTCGGTGGTAGGT
POU5F1B-F	GTGTTCAGCCAAAAGACCATCT
POU5F1B-R	GGCCTGCATGAGGGTTTCT
ChIP-ALP-F	GCTTCTTCTTGCGGTAGCCAG
ChIP-ALP-R	TGTCTTTACCGTCGGCATCTTCC
ChIP-BMP2-F	ACTCATCGTGCAGCAGCTCT
ChIP-BMP2-R	AGCTCAGAGGCAGGCGATTA
MYC-shRNA	CTGCGACGAGGAGGAGAACTT
