# Supplementary material for: MYC promotes fibroblast osteogenesis by regulating ALP and BMP2 to participate in ectopic ossification of ankylosing spondylitis
Source: Arthritis Res Ther. 2023 Feb 21;25:28. doi: 10.1186/s13075-023-03011-z (PMC9942334; doi:10.1186/s13075-023-03011-z)
Supplement: Supplementary file 2 — Additional file 2:Supplementary file 2. [file 13075_2023_3011_MOESM2_ESM.pdf]

Original Gels and Blots

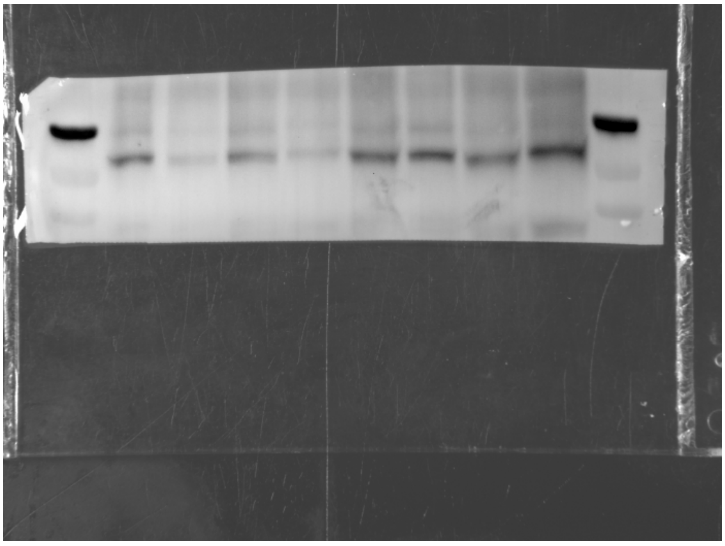

Figure (1). Original picture of figure 1c (MYC) in the main figures: Lane 1 to 10 (from left to right) are protein marker, OA samples 1- 4, AS samples 1- 4, and protein marker.

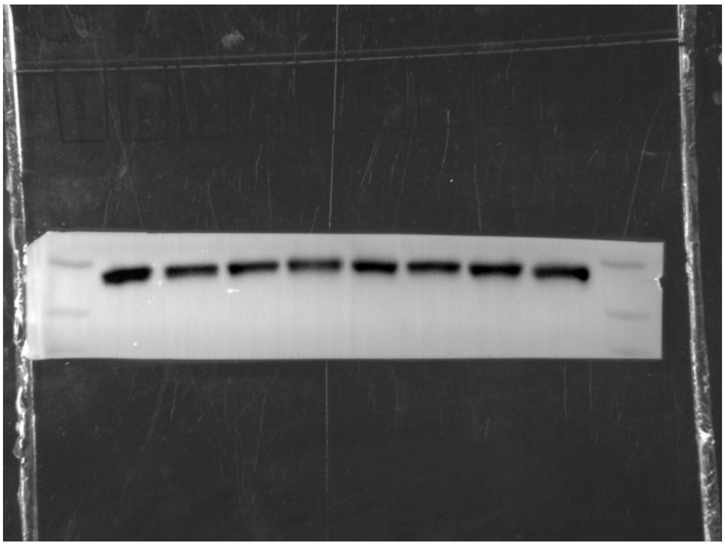

Figure (2). Original picture of figure 1c (GAPDH) in the main figures: Lane 1 to 10 (from left to right) are protein marker, OA samples 1-4, AS samples 1-4, and protein marker.

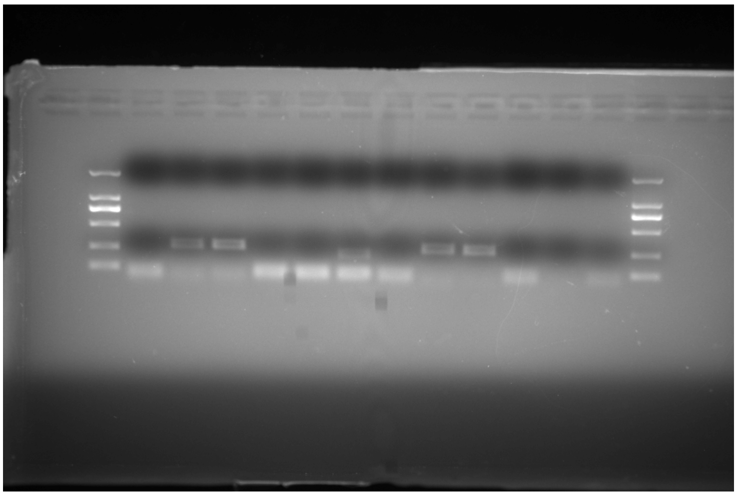

Figure (3). Original picture of figure 2d (ALP) in the main figures: Lane 1 to 4 (from left to right) are DNA ladder, IgG, MYC, and Input. The other lanes are samples not mentioned in this article.

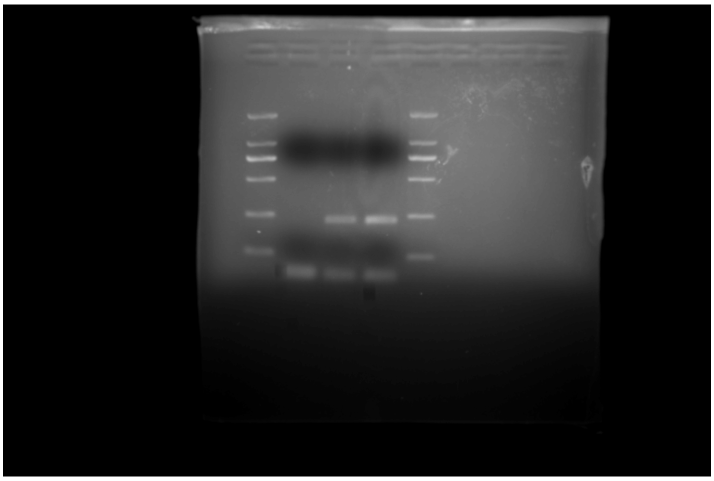

Figure (4). Original picture of figure 2d (BMP2) in the main figures: Lane 1 to 5 (from left to right) are DNA ladder, IgG, MYC, Input, and DNA ladder.
